# miR‐122‐5p modulates the radiosensitivity of cervical cancer cells by regulating *cell division cycle 25A* (*CDC25A*)

**DOI:** 10.1002/2211-5463.12730

**Published:** 2019-09-29

**Authors:** Feng‐Na Ding, Bao‐Hong Gao, Xia Wu, Chun‐Wu Gong, Wei‐Qing Wang, Shu‐Mao Zhang

**Affiliations:** ^1^ Department of Gynaecology Linyi Cancer Hospital Shandong China; ^2^ Department of Gynaecology The Third People's Hospital of Linyi Shandong China; ^3^ Department of Oncology The Third People's Hospital of Linyi Shandong China; ^4^ Department of Radiology The Third People's Hospital of Linyi Shandong China; ^5^ Department of Radiology Linyi Cancer Hospital Shandong China

**Keywords:** *CDC25A*, cervical cancer, miR‐122‐5p, radiosensitivity

## Abstract

Cervical cancer is one of the most common gynecological malignancies globally, Unfortunately, radiotherapy and chemotherapy are not effective at treating some cases of this disease, and the 5‐year survival rate is only 40–50%. *Cell division cycle 25A* (*CDC25A*) has been shown to induce radioresistance in a variety of tumor cells, but the role of *CDC25A* in the radioresistance of cervical cancer has not been fully elucidated. Here, we report that *CDC25A* is highly expressed and miR‐122‐5p lowly expressed in cervical cancer tissues and cells. The TargetScan database was used to predict *CDC25A* as a target of miR‐122‐5p, and the interactions between miR‐122‐5p and *CDC25A* were further confirmed by western blot, real‐time PCR and dual‐luciferase reporter assay. Under X‐ray irradiation, up‐regulation of *CDC25A* can promote the radiation resistance of cervical cancer cells, whereas overexpression of miR‐122‐5p or knockdown of *CDC25A* inhibits the survival and induces apoptosis of cervical cancer colonies. In conclusion, our data suggest that miR‐122‐5p enhances the radiosensitivity of cervical cancer cells by targeting *CDC25A*.

**Abbreviations**
3′ UTR: 3′ untranslated region
CCK‐8: Cell Counting Kit‐8
*CDC25A*: *cell division cycle 25A*
IHC: immunohistochemistry
miRNA: microRNA
MT: mutant type
NC: negative control
qRT‐PCR: quantitative RT‐PCR
SD: standard deviation
WT: wild‐type

As one of the most common gynecological malignancies in the world, cervical cancer has been a major health problem for women, with its morbidity and mortality increasing year by year 1, 2, 3. So far, surgery, radiotherapy and chemotherapy are still mainly used for the treatment of patients with cervical cancer 4, 5. Unfortunately, radiotherapy and chemotherapy are not effective in some cases, and the 5‐year survival rate of this disease is only 40–50% 6. Even more serious, it has been increasingly reported that some tumor cells have reduced sensitivity to X‐rays, and the treatment effect for patients with cervical cancer is far from meeting our expectations 7, 8, 9. Therefore, it is urgent to explore the mechanism of radiation resistance of cervical cancer cells and improve the prognosis of patients.

The *cell division cycle 25* (*CDC25*) family members are important modulators of some steps in the cell cycle, including the activation of several cyclin‐dependent kinases 10. Previous studies have reported that *CDC25A* is a common indicator for the diagnosis of non‐small‐cell lung cancer, and its high expression often indicates poor prognosis 11, 12. Some studies have pointed out that *CDC25A* is involved in the regulation of the cell cycle and induces synthesis of radiation‐resistant DNA to decrease radiosensitivity of the cells 10, 13. *CDC25A* has been shown to induce radioresistance in a variety of tumor cells, such as non‐small‐cell lung cancer, esophageal cancer and colon cancer 14, 15, 16, 17. However, the role of *CDC25A* in the radioresistance of cervical cancer and its mechanism has not been fully elucidated.

MicroRNAs (miRNAs) are a class of small noncoding RNAs that contain 18–24 nucleotides with post‐transcriptional regulation of translational inhibition or degradation by specific binding to the 3′ untranslated region (3′ UTR) of the target gene 18, 19. Studies have reported that some miRNAs are involved in regulating the radiation response of different cancer cells to increase radiosensitivity or radiotherapy resistance 20, 21, 22, 23. For example, miR‐132 and miR‐18a increase the radiosensitivity of cervical cancer cells 24, 25, whereas miR‐208a increases the radioresistance of lung cancer cells 26. However, the role and molecular mechanism of miR‐122‐5p in the radiation resistance of cervical cancer cells remain unclear.

More and more studies have shown that the expression of *CDC25A* is regulated by multiple miRNAs, and it participates in the radiation resistance of various tumor cells. For example, miR‐365 promotes the radiosensitivity of non‐small‐cell lung cancer cells by targeting the regulation of *CDC25A* expression 14. In addition, in prostate cells, studies have indicated that miR‐449a enhances radiosensitivity of tumor cells by modulating *CDC25A* 27. It is reported that overexpression of miR‐122‐5p in hepatocytes inhibits proliferation and promotes apoptosis of cells 28. Through bioinformatics analysis, we found that *CDC25A* is a potential target gene of miR‐122‐5p, which prompted us to investigate whether miR‐122‐5p can regulate the expression of *CDC25A* and its relationship with the radiotherapy resistance of cervical cancer cells.

The aim of this study was to investigate the expression of miR‐122‐5p and *CDC25A* in cervical cancer cells and their role as regulatory mechanisms in the radiosensitivity of cervical cancer cells. miR‐122‐5p and *CDC25A* are expected to further elucidate the potential treatment for patients with cervical cancer and improve prognosis.

## Materials and methods

### Patient specimens

All patients in this experiment signed an informed consent, and the experimental program was approved by the Clinical Ethics Committee of Linyi Cancer Hospital.

All experiments were conducted in accordance with the ethical guidelines of the World Medical Association (Declaration of Helsinki). We selected 77 cervical cancer cases with fresh cancer and paracancerous tissue samples, and the paracancerous tissues were confirmed by pathology as normal cervical mucosa from 2015 to 2018. All patients had complete clinical and pathological data, and the pathological results of paraffin specimens were confirmed by professional pathologists. Patients diagnosed with active infection, human papillomavirus infection or chronic inflammatory disease were excluded from our study.

### Immunohistochemistry

The wax block containing the cervical cancer and the adjacent tissues was sliced, and the xylene was used for dewaxing and hydrating. They were incubated for 30 min at room temperature with a 0.3% H_2_O_2_ solution, overnight at 4 °C with primary antibody (anti‐CDC25A; ab2357, 1 : 100; Abcam, Shanghai, China) and for 1 h at 37 °C with second antibody (goat anti‐rabbit IgG). The sections were then rinsed with PBS buffer. 2,4‐Diaminobutyric acid (Hubei Baiaosi Bioscience Co., Ltd. Wuhan, China) was then used to stop the reaction after color development. The scoring criteria for immunohistochemistry (IHC) were completed by the pathologists from our hospital.

### Cell Culture

Overexpression plasmids, short hairpin RNA (shRNA) control, miRNA control, miR‐122‐5p mimics (5′‐UGGAGUGACAAUGGUGUUUG‐3′) and miR‐122‐5p inhibitor (5′‐CAAACACCAUUGUCACACUCCA‐3′) were purchased from RiboBio (Guangzhou, China). The construct of the plasmid that included *CDC25A* 3′ UTR was purchased from RiboBio. Human cervical cancer cell lines (HeLa, HeLa229, C‐33A, CaSki, ME180 and SiHa) and normal cervical epithelial cells (END1/E6E7) were purchased from Shanghai Kanglang Biotechnology Co., Ltd. Shanghai, China. All cells were cultured in RPMI 1640 medium (Gibco, Grand Island, NY, USA), containing 10% FBS (Gibco) and 1% double antibody (penicillin/streptomycin; Gibco, Thermo Fisher Scientific, Waltham, MA, USA) at 5% CO_2_, 37 °C.

### Western blotting

The cells were rinsed with PBS and lysed with radioimmunoprecipitation assay lysis buffer containing protease inhibitor (Thermo Science, Rockford, IL, USA). The supernatant was collected after high‐speed centrifugation and heated in a water bath to denature the protein. After quantifying the protein by the bicinchoninic acid method, proteins were separated by SDS/PAGE and transferred to a nitrocellulose membrane (Millipore Corp, Billerica, MA, USA). Then, the membrane was blocked with skim milk powder for 30 min at 37 °C, 5% CO_2_, and incubated overnight with primary antibody, anti‐CDC25A (ab2357; Abcam), at 4 °C. The poly(vinylidene difluoride) membrane was rinsed with the TBS+Tween solution and then incubated with the secondary antibody (Hubei Baiaosi Bioscience Co., Ltd., 1 : 2000) for 1 h at room temperature, and chemiluminescence was developed using a hypersensitive enhanced chemiluminescent (Hubei Baiaosi Bioscience Co., Ltd.). β‐Actin was used as an internal reference.

### Quantitative RT‐PCR assay

Total RNA in cervical cancer tissues and cells was extracted using TRIzol (Invitrogen, Carlsbad, CA, USA) according to the instructions. RNA was synthesized into cDNA using a prime Script RT kit (Takara Bio, Inc., Otsu, Japan). The expression of miR‐122‐5p and CDC25A was detected using ABI 7500 Real‐Time PCR system (Applied Biosystems, Foster City, CA, USA) and the SYBR Green PCR kit (Takara, Dalian, China). U6 was used as an internal reference. Specific PCR primers were synthesized by Thermo Fisher Scientific, Inc. The primer sequence was as follows: miR‐122‐5p: 5′‐UGGAGUGUGACAAUGGUGUUUG‐3′; CDC25A: forward, 5′‐ATGAGGATGATGGCTTCG‐3′, reverse, 5′‐AACACTGACCGAGTGCTG‐3′; U6: forward: 5′ CTCGCTTCGGCAGCACA 3′, reverse: 5′ AACGCTTCACGAATTTGCGT 3′. The relative expression was calculated using the $2^{\Delta \Delta C_{t}}$ method. Transfection validity was detected 48 h later.

### Transfection

*Cell division cycle 25A* (*CDC25A*) overexpression plasmid, *CDC25A* shRNA, control plasmid and control shRNA were screened and constructed by Shanghai GeneChem (Shanghai, China). Cells were seeded in 96‐well plates at 4 × 10^4^ per well. CDC25A overexpression plasmid and control plasmid were transfected into HeLa cells using Lipofectamine 2000 (Invitrogen) according to the manufacturer's protocols. CDC25A shRNA and control shRNA were transfected into SiHa, respectively. Transfection validity was detected 48 h later.

### Radiation

Cells were radiated with different radiation doses (0, 2, 4, 6 and 8 Gy) using an X‐RAD 320 X‐ray radiator (Softex, Goyang, Korea) at a dose rate of 2 Gy·min^−1^.

### Cell Counting Kit‐8 assay

HeLa and SiHa cells in the logarithmic growth phase were selected and seeded in 96‐well plates at 2000 cells per well. The cells were radiated with different radiation doses, and after incubation for 48 h, 10 μL of Cell Counting Kit‐8 (CCK‐8) solution (Sigma‐Aldrich, St. Louis, MO, USA) was added to measure the absorbance at 450 nm on a microplate reader (Bio‐Rad Laboratories, Inc., Hercules, CA, USA), which indicates the ability of cell proliferation. Each group of experiments was repeated three times.

### Colony formation assay

Cervical cancer cells were seeded in 24‐well plates at 2 × 10^3^ cells per well. Then they were treated with X‐rays at a dose of 4 Gy, incubated at 37 °C for 14 days and fixed with 4% paraformaldehyde for 10 min. One milliliter of 10% crystal violet was added to each well for staining. Then, the colonies were rinsed three times with PBS, and the number of colonies formed was recorded under a microscope.

### Flow cytometry analysis of cell apoptosis

After transfection, the cells were cultured for 24 h under irradiation with 4 Gy, and harvested and centrifuged to remove the supernatant at a rate of 500 ***g*** Apoptosis ratio was assessed by flow cytometry (FACSCalibur; BD Biosciences, Franklin Lakes, NJ, USA) according to instructions of the Annexin V/FITC Apoptosis Kit (Sigma‐Aldrich).

### Dual‐luciferase reporter gene assay

It was predicted by TargetScan (http://www.targetscan.org) that there is a base‐complementary pairing relationship between miR‐122‐5p and *CDC25A*. Cervical cancer cells were seeded in 96‐well plates and transfected with miR‐122‐5p mimics + *CDC25A*‐wild‐type (WT), miR‐122‐5p mimics + *CDC25A*‐mutant type (MT), miR‐122‐5p negative control (NC) + CDC25A‐WT and miR‐122‐5p NC + *CDC25A*‐MT. After 72 h of transfection, cervical cancer cells were collected and assayed for luciferase activity according to the instructions of the dual‐luciferase reporter assay kit (Promega, Shanghai, China).

### Statistical analysis

The statistical processing software used in this study was graphpad prism 7 (San Diego, CA, USA). All data were expressed as mean ± standard deviation (SD). The *t*‐test was used to compare the measurement data between the two groups. The χ^2^ test and logistic regression analysis were used to analyze the correlation between the expression of CDC25A and the clinicopathological parameters of cervical cancer. A *P*‐value <0.05 was considered statistically significant.

## Results

### CDC25A was highly expressed in cervical cancer tissues and cells, whereas miR‐122‐5p was lowly expressed

**Figure 1:**
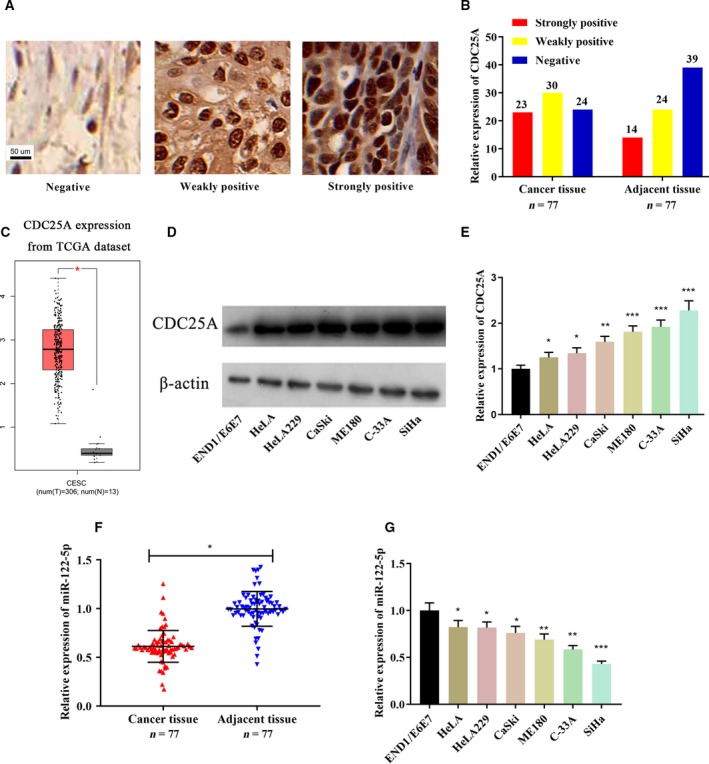
CDC25A was significantly up‐regulated and miR‐122‐5p was significantly down‐regulated in cervical cancer tissues and cells. The representative IHC images of the three levels of staining for CDC25A (strongly positive, weakly positive and negative) are shown in (A). (B) The number of strongly positive, weakly positive and negative expressions of CDC25A in 77 cases of cervical cancer and adjacent tissues was counted. (C) The expression levels of CDC25A in cervical cancer and normal tissues were compared using The Cancer Genome Atlas (TCGA) public dataset. (D, E) The expression of CDC25A in normal cervical epithelial cells (END1/E6E7) and six cervical cancer cell lines (HeLa, HeLa229, C‐33A, CaSki, ME180 and SiHa cells) was detected by western blot (*n* = 3). (F) qRT‐PCR was used to detect the expression of miR‐122‐5p in cervical cancer tissues and adjacent tissues (*n* = 3). (G) The expression of miR‐122‐5p in six cervical cancer cells and normal cervical epithelial cells was detected by qRT‐PCR (*n* = 3). Error bars represent SD. Comparisons between groups were analyzed using *t*‐tests (two‐sided). *P <* *0.05, **P <* *0.01, ***P <* *0.001. Scale bar: 50 μm. CESC, cervical squamous cell carcinoma.

To investigate whether the radiotherapy resistance of cervical cancer cells is related to the expression level of CDC25A, we first detected by IHC the expression level of CDC25A in cancer tissues and adjacent tissues from 77 patients with cervical cancer. The representative IHC images of the three levels of staining for CDC25A (strongly positive, weakly positive and negative) are shown in Fig. 1A. The statistical results showed that the positive rate of CDC25A in cervical cancer tissues was significantly increased compared with that of adjacent tissues (χ^2^ = 6.4273; *P *=* *0.0402; Fig. 1B). The expression levels of CDC25A in cervical cancer and normal tissues were compared using The Cancer Genome Atlas public dataset (http://gepia.cancer‐pku.cn/), which showed that the expression of CDC25A in cervical cancer tissues was significantly higher (Fig. 1C). The expression of CDC25A in different cervical cancer cells was detected by western blot. The results showed that the expression of CDC25A was significantly increased in six kinds of cervical cancer cells (HeLa, HeLa229, C‐33A, CaSki, ME180 and SiHa) compared with that of normal cervical epithelial cells (Fig. 1D,E). We also detected the expression level of miR‐122‐5p in cervical cancer tissues and adjacent normal tissues by quantitative RT‐PCR (qRT‐PCR). It was found that miR‐122‐5p was significantly decreased in cervical cancer tissues compared with that of adjacent tissues (Fig. 1F). Consistent with the clinical sample results, the expression levels of miR‐122‐5p were significantly decreased in six cervical cancer cells compared with that of normal cervical epithelial cells (Fig. 1G).

### The expression level of CDC25A was related to multiple clinical pathological indicators of patients with cervical cancer

Subsequently, χ^2^ test and logistic regression analysis were used to analyze the relationship between the expression level of *CDC25A* and the clinicopathological indicators of patients with cervical cancer. We found that the higher expression of *CDC25A* in cervical cancer tissues was related to more advanced clinical stage and earlier lymph node metastasis, but not to age, tumor volume and CA125 level (Table 1).

**Table 1:** The relationship between *CDC25A* expression and clinical pathological factors in cervical cancer.

Pathological indicators	No. of patients	CDC25A relative expression		Pearson χ^2^ test		Logistic regression analysis	
		High expression	Low expression	*F*‐value	*P*‐value	Exp(B)	*P*‐value
Age, years							
≥ 60	42	31	11	1.0674	0.3015	0.935	0.316
< 60	35	22	13				
Tumor size, d·cm^−1^							
< 3	39	23	16	3.5788	0.0585	1.977	0.076
≥ 3	38	30	8				
Clinical stage							
I + II	34	28	6	5.1888	0.0227	5.431	0.032
III + IV	43	25	18				
CA125 level (μg·L^−1^)							
< 35	34	20	14	2.822	0.0918	1.211	0.104
≥ 35	43	33	10				
Lymph node metastasis							
No	38	22	16	4.1827	0.0408	2.932	0.048
Yes	39	31	8				

### *CDC25A* regulated the sensitivity of cervical cancer cells to radiation

**Figure 2:**
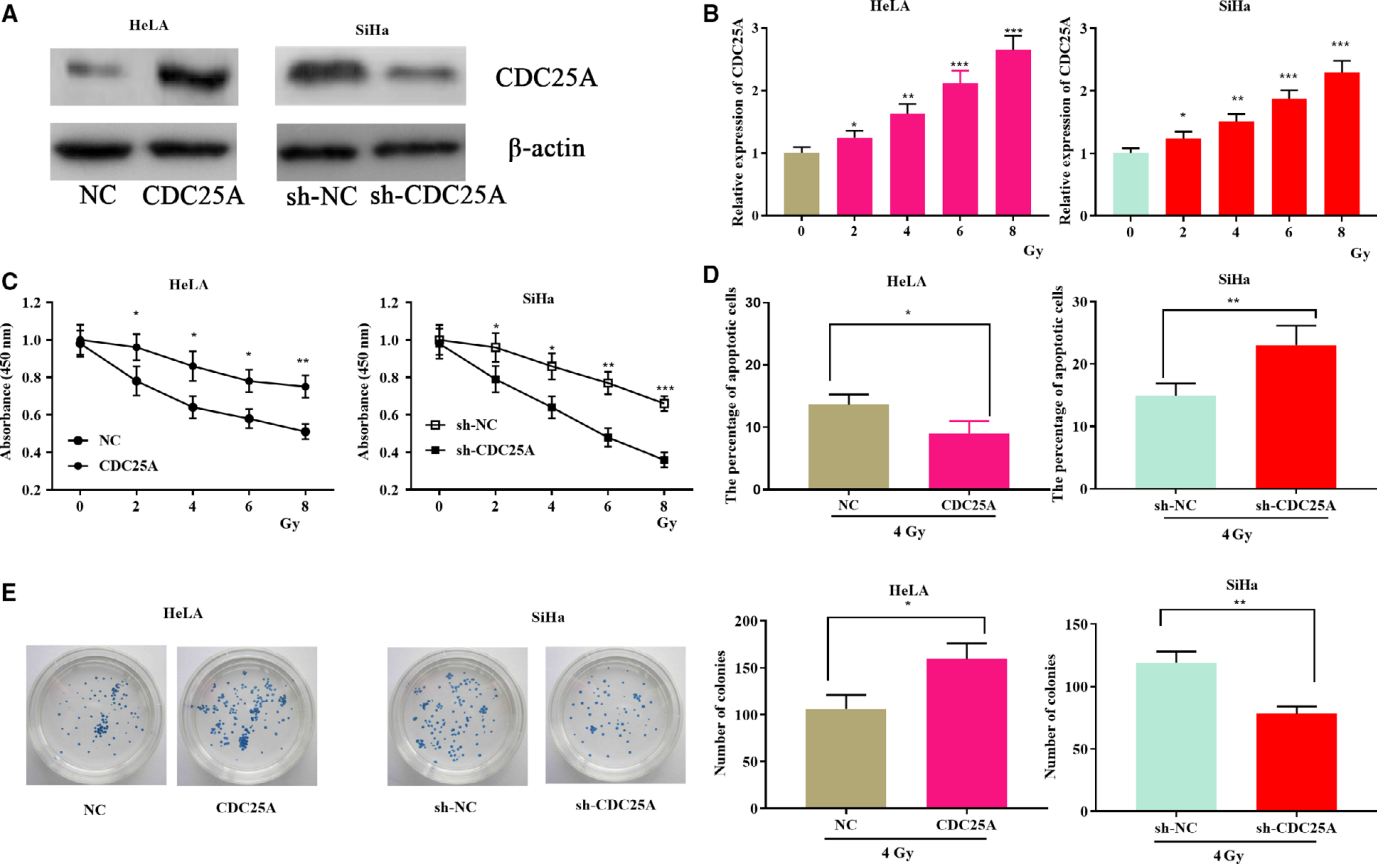
*CDC25A* reduces the radiosensitivity of cervical cancer cells. (A) Cells models with overexpressed CDC25A (left) and underexpressed CDC25A (right) were successfully constructed. (B) Expression levels of HeLa and SiHa cells at different doses of radiation were detected (*n* = 3). (C) CCK‐8 was used to evaluate the activity of cervical cancer cells when being exposed to irradiation (*n* = 3). (D) Flow cytometry was used to evaluate the percentage of apoptotic cervical cancer cells under irradiation (*n* = 3). (E) Colony formation assay was used to evaluate the cell proliferation under irradiation (*n* = 3). Error bars represent SD. Comparisons between groups were analyzed using *t*‐tests (two‐sided). *P <* *0.05, **P <* *0.01, ***P < 0.001.

Notably, among the six types of cervical cancer cells, we found that the expression level of *CDC25A* in SiHa cells was the highest, whereas the expression level of HeLa cells was the lowest. Therefore, SiHa cells were selected to construct the knockdown model, and HeLa cells were used to construct the overexpression model. Next, to study the regulation of *CDC25A* on the sensitivity of cervical cancer cells to radiotherapy, HeLa and SiHa cells were selected for further experiments (Fig. 2A). The expression of CDC25A in cervical cancer cells was detected by western blot at different radiation doses. The results showed that the expression of CDC25A in HeLa and SiHa cells was significantly increased under X‐ray irradiation with doses of 2, 4, 6, and 8 Gy compared with that with non‐ray (0 Gy) irradiation (Fig. 2B). In addition, the CCK‐8 assay was used to detect the activity of cervical cancer cells at different doses of radiation. The results showed that HeLa cells with overexpressed *CDC25A* were more resistant to radiation than was the control group. In contrast, SiHa cells with *CDC25A* knockdown were more sensitive to X‐rays (Fig. 2C). According to previous studies 19, we chose the irradiation dose of 4 Gy to perform functional assays. Flow cytometry showed that the apoptosis proportion of HeLa cells with *CDC25A* overexpressed was significantly reduced compared with that of the control group at a dose of 4 Gy. The apoptosis proportion of SiHa cells with CDC25A knockdown was significantly increased (Fig. 2D). Subsequently, we measured the number of colonies formed of cervical cancer cells at a dose of 4 Gy. The results showed that the number of colonies formed by HeLa cells with *CDC25A* overexpressed was more than that of the control group, whereas the number of colonies formed by SiHa cells with CDC25A knockdown was significantly lower than that of the control group (Fig. 2E). Collectively, these results suggest that *CDC25A* can reduce the sensitivity of cervical cancer cells.

### *CDC25A* was a direct target of miR‐122‐5p

**Figure 3:**
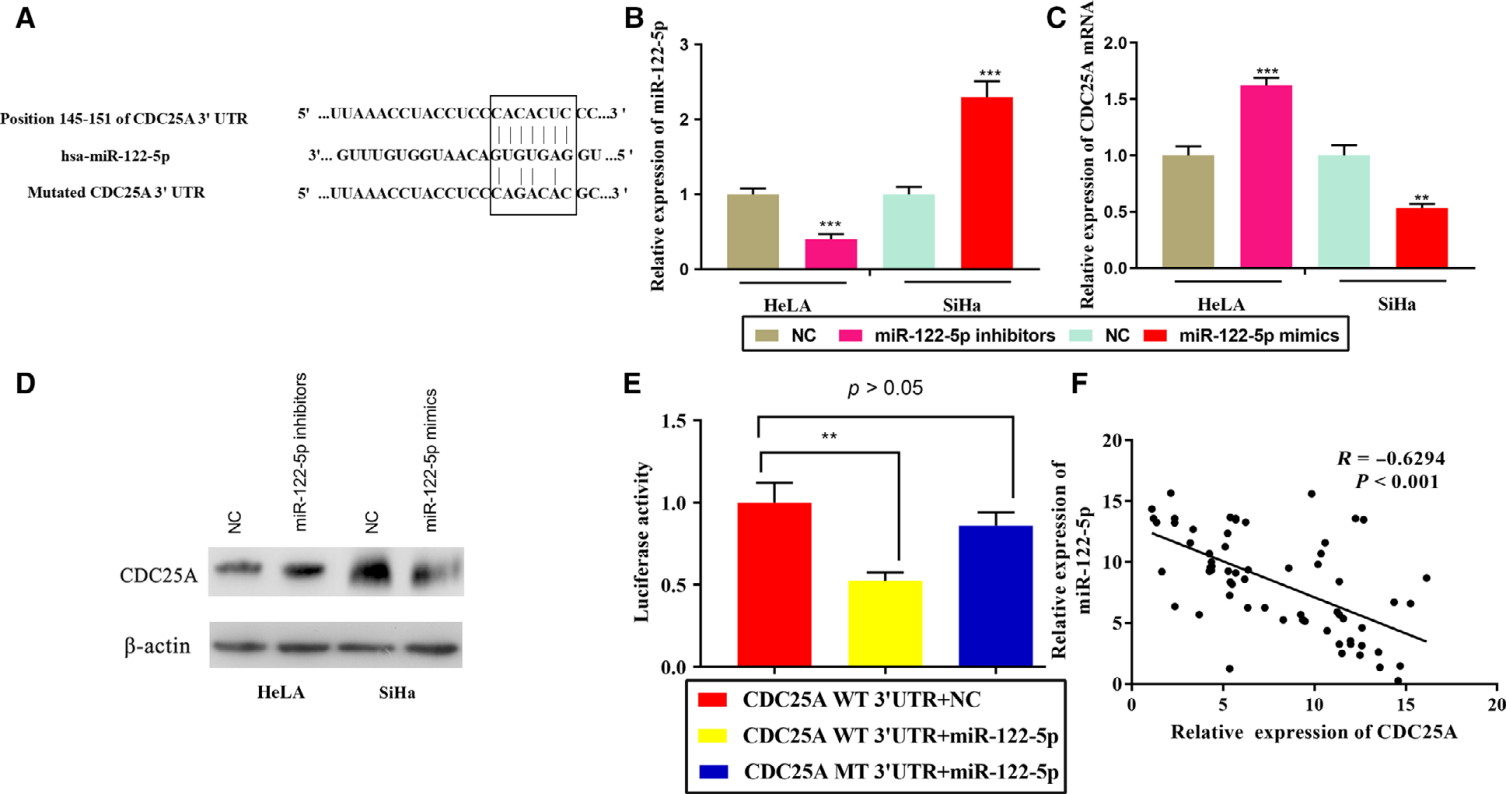
Targeting relationship between miR‐122‐5p and *CDC25A*. (A) Prediction of the binding site between miR‐122‐5p and *CDC25A* by TargetScan. (B, C) qRT‐PCR was used to detect the expression of miR‐122‐5p and CDC25A mRNA in cervical cancer cells (*n* = 3). (D) Western blot was used to detect the expression of CDC25A in cervical cancer cells (*n* = 3). (E) WT *CDC25A* + miR‐122‐5p, mutant type CDC25A + miR‐122‐5p and WT CDC25A + NC were transfected into 293T cells, and the three luciferase activities were compared (*n* = 3). (F) There was a negative correlation between miR‐122‐5p and CDC25A expression in 77 patients with cervical cancer (Spearman's correlation, *P* < 0.0001, *R* = −0.6294). Error bars represent SD. Comparisons between groups were analyzed using *t*‐tests (two‐sided). *P < 0.05, **P < 0.01, ***P < 0.001.

miRNAs function primarily through base pairing with complementary sequences to the target region. Subsequently, the potential target of miR‐122‐5p was predicted using the TargetScan Bioinformatics database, and it was found that *CDC25A* is a candidate target gene for miR‐122‐5p (Fig. 3A and Table S1). Compared with the control group, the expression level of miR‐122‐5p in HeLa cells transfected with miR‐122‐5p inhibitors was significantly reduced. On the other hand, miR‐122‐5p expression was significantly increased in SiHa after transfection with miR‐122‐5p mimics (Fig. 3B). RT‐PCR and western blot analysis showed that transfection of miR‐122‐5p inhibitors in HeLa cells caused an increase in *CDC25A* expression, whereas transfection of miR‐122‐5p mimics in SiHa cells caused a decrease in CDC25A expression (Fig. 3C,D). Subsequently, luciferase reporter gene verification experiments revealed that miR‐122‐5p binds to the 3′ UTR of *CDC25A* (Fig. 3E), and its expression negatively correlated with *CDC25A* expression in clinical samples (Fig. 3F).

### miR‐122‐5p promoted the sensitivity of cervical cancer cells by inhibiting the expression of *CDC25A*

**Figure 4:**
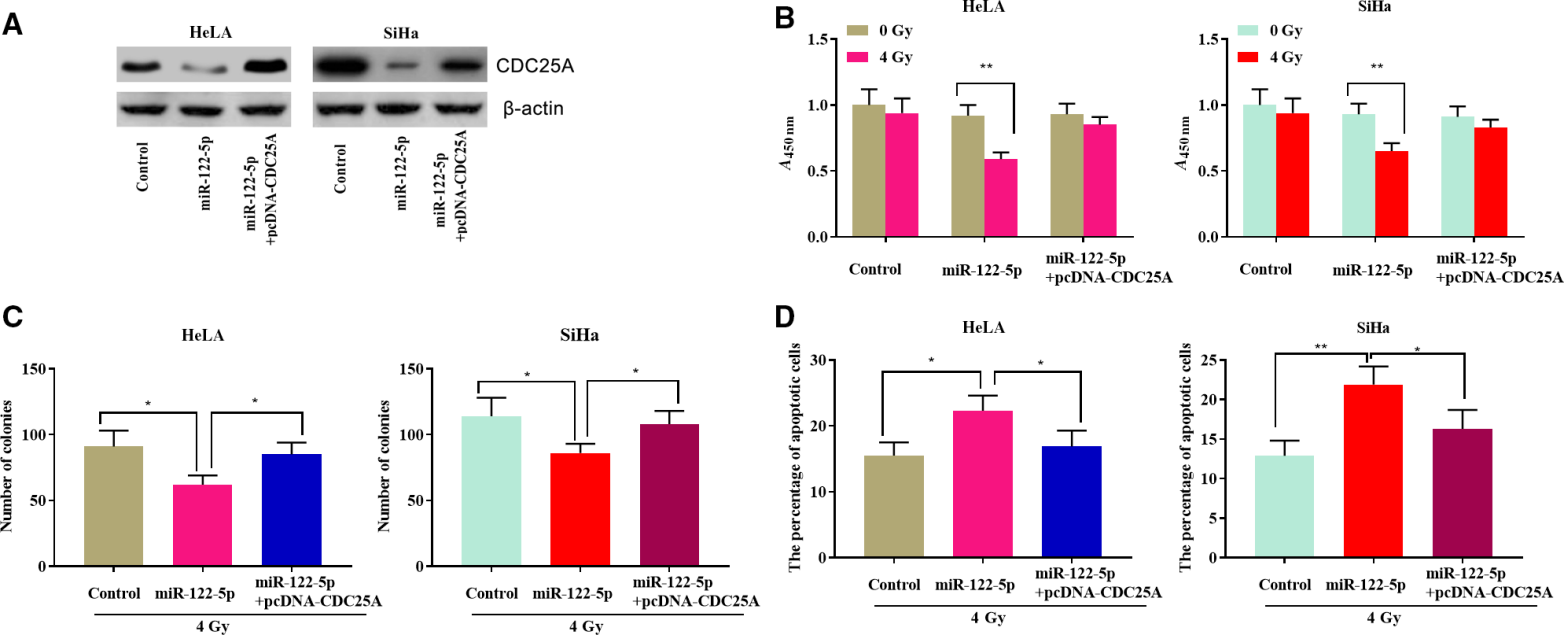
miR‐122‐5p enhances the radiosensitivity of cervical cancer cells by modulating CDC25A. (A) Western blot was used to detect the expression level of CDC25A after transfection (*n* = 3). (B) After being treated with 4 Gy ionizing radiation, the cell viability of transfected HeLa and SiHa cells was examined (*n* = 3). (C) After being exposed to irradiation (4 Gy), the survival of cervical cancer cells was detected by colony formation assay (*n* = 3). (D) After being exposed to irradiation (4 Gy), the percentage of apoptotic cervical cancer cells was detected by flow cytometry (*n* = 3). Error bars represent SD. Comparisons between groups were analyzed using *t*‐tests (two‐sided). *P < 0.05, **P < 0.01, ***P < 0.001.

To further elucidate whether miR‐122‐5p and *CDC25A* modulate the radiosensitivity of HeLa and SiHa cells, control miRNA, miR‐122‐5p mimics and miR‐122‐5p‐pcDNA‐CDC25A were transfected into HeLa and SiHa cells, respectively. Western blot indicated that pcDNA‐CDC25A was able to reverse the low levels of CDC25A caused by overexpression of miR‐122‐5p, indicating that the transfection assay was successful (Fig. 4A). The CCK‐8 assay was performed at X‐ray irradiation with a dose of 4 Gy, and high expression of *CDC25A* in HeLa and SiHa cells significantly reversed the decrease in proliferation induced by miR‐122‐5p (Fig. 4B). Subsequently, a colony formation assay was performed under X‐ray irradiation at a dose of 4 Gy. The results indicated that overexpression of *CDC25A* in HeLa and SiHa impaired the decrease in the number of colonies caused by miR‐122‐5p (Fig. 4C). Finally, an apoptosis experiment was performed under X‐ray at a dose of 4 Gy, and *CDC25A* reduced the increase in apoptotic rate caused by miR‐122‐5p (Fig. 4D).

## Discussion

Surgery and chemotherapy combined with radiotherapy is currently the main treatment for cervical cancer. In recent years, with the advancement and development of radiotherapy technology, its role in the clinic has become increasingly prominent 29, 30, 31. Unfortunately, the therapeutic effect of radiotherapy has not met our expectations yet, and the reduced sensitivity of cancer cells to radiotherapy is an important cause of recurrence and metastasis in some patients 7, 32. Obviously, it is necessary to explore the resistance mechanism of cervical cancer cells to radiation therapy and provide potential new therapeutic targets for patients with radiotherapy resistance.

In cancer cells, DNA damage, cell cycle checkpoints and abnormal expression of important genes were involved in the radioresistance of tumor cells 33, 34. *CDC25A* modulates crucial transitions between cell cycle phases during cell division, and in the case of DNA damage, it belongs to the key targets of the checkpoint machinery that ensure genetic stability 35. Studies have shown that increased expression of *CDC25A* was associated with radiation resistance of tumor cells, such as advanced esophageal squamous cell carcinoma and non‐small‐cell lung cancer 14, 15, 16. In this study, by analyzing the correlation between the expression levels of miR‐122‐5p and *CDC25A* in the cancer tissues of 77 patients with cervical cancer, we found a negative correlation between them, suggesting a potential regulatory relationship between them. We successfully constructed cell models with overexpressed *CDC25A* and lowly expressed *CDC25A*, and found that overexpression of *CDC25A* can significantly improve the resistance of cervical cancer cells to radiotherapy, promote the survival of cervical cancer cell colonies and inhibit apoptosis. Knockdown of *CDC25A* can increase the sensitivity of cervical cancer cells to radiotherapy, inhibit the survival of cervical cancer cell colonies and promote apoptosis. These results are consistent with previous reports, and we conclude that *CDC25A* can induce radiotherapy resistance in cervical cancer cells. In this study, we also demonstrated that *CDC25A* was one of the target genes of miR‐122‐5p.

More and more studies have shown that the reduced sensitivity of tumor cells to X‐rays is associated with abnormal expression of miRNAs 36, 37. As previously reported, miRNAs are associated with radiosensitivity in a variety of tumors. For example, miR‐205 improves radiation sensitivity of prostate cancer cells by impairing DNA damage repair through protein kinase Cε and ZEB1 inhibition 38, and miR‐153‐3p enhances the radiosensitivity of human glioma cells by targeting B cell lymphoma‐2 21. Furthermore, miR‐16‐5p enhances the radiosensitivity of prostate cancer cells by modulating the cyclin D1/E1‐pRb‐E2F1 signaling pathway 39. miR‐122 induces radiosensitization in non‐small‐cell lung cancer cell lines and breast cancer 40, 41. In this study, we demonstrated that compared with that of adjacent tissues, the positive rate of highly expressed *CDC25A* in cancer tissues was significantly higher, whereas miR‐122‐5p was significantly lowly expressed. We also confirmed that *CDC25A* is a target of miR‐122‐5p by the dual‐luciferase reporter gene. Thus, we confirmed that miR‐122‐5p can inhibit the expression of CDC25A, thereby promoting the radiosensitivity of cervical cancer cells.

## Conclusions

This study confirmed that *CDC25A* can induce radiation resistance in cervical cancer cells, and that the expression of *CDC25A* is negatively regulated by miR‐122‐5p. We described one of the reasons for the sensitivity of cervical cancer cells to radiotherapy, which can help improve the prognosis of patients with cervical cancer, and potential therapeutic targets were thus provided. However, this study was limited to cell experiments, and thus requires animal experiments and a larger sample size to confirm the earlier conclusions.

## Conflict of interest

The authors declare no conflict of interest.

## Author contributions

F‐ND and S‐MZ conceived and designed the experiments. F‐ND, B‐HG, XW, C‐WG, W‐QW and S‐MZ performed the experiments. S‐MZ performed the statistical analysis. F‐ND, B‐HG and S‐MZ wrote the paper. All authors read and approved the final manuscript.

## Supporting information


**Table S1**. Using TargetScan database to predict downstream targets of microRNA‐122‐5p.
